# Research progress on the fanconi anemia signaling pathway in non-obstructive azoospermia

**DOI:** 10.3389/fendo.2024.1393111

**Published:** 2024-05-23

**Authors:** Haohui Xu, Yixin Zhang, Caiqin Wang, Zhuoyan Fu, Jing Lv, Yufang Yang, Zihan Zhang, Yuanmin Qi, Kai Meng, Jinxiang Yuan, Xiaomei Wang

**Affiliations:** ^1^Lin He’s Academician Workstation of New Medicine and Clinical Translation, Jining Medical University, Jining, China; ^2^College of Second Clinical Medical, Jining Medical University, Jining, China; ^3^College of Clinical Medicine, Jining Medical University, Jining, China; ^4^College of Mental Health, Jining Medical University, Jining, China; ^5^College of Basic Medicine, Jining Medical University, Jining, China

**Keywords:** non-obstructive azoospermia, Fanconi anemia pathway, Fanconi anemia gene, interstrand crosslinks, homologous recombination

## Abstract

Non-obstructive azoospermia (NOA) is a disease characterized by spermatogenesis failure and comprises phenotypes such as hypospermatogenesis, mature arrest, and Sertoli cell-only syndrome. Studies have shown that FA cross-linked anemia (FA) pathway is closely related to the occurrence of NOA. There are FA gene mutations in male NOA patients, which cause significant damage to male germ cells. The FA pathway is activated in the presence of DNA interstrand cross-links; the key step in activating this pathway is the mono-ubiquitination of the FANCD2-FANCI complex, and the activation of the FA pathway can repair DNA damage such as DNA double-strand breaks. Therefore, we believe that the FA pathway affects germ cells during DNA damage repair, resulting in minimal or even disappearance of mature sperm in males. This review summarizes the regulatory mechanisms of FA-related genes in male azoospermia, with the aim of providing a theoretical reference for clinical research and exploration of related genes.

## 1 Introduction

Male infertility is a common reproductive disease that affects multiple families eager to create a new life. Existing research statistics have found that approximately seven out of every 100 males suffer from infertility symptoms (1). Of these, 13-17% of infertile men are due to azoospermia, which accounts for 55-65% of non-obstructive azoospermia (2). It is generally believed that the main cause of male infertility may be abnormal reduction in sperm count or even azoospermia caused by factors such as congenital inheritance or acquired living environment (3). Azoospermia is an extreme form of decreased sperm count during ejaculation, divided into two types: obstructive azoospermia (OA) and non-obstructive azoospermia (NOA) (4). The viability of sperm is closely related to the health status of the testes, and NOA is the result of abnormal gene expression in the testes. The entire ejaculatory duct of NOA is open and functioning normally, but the number of healthy sperm is very low, which may be related to genetic mutations (5). Interstrand cross-linking (ICL) has a direct effect on the proliferation of male germ cells and sperm maturation, and the error repair of ICL will lead to the production of NOA (6). Fanconi anemia (FA) is a chromosomally inherited disease caused by mutations in the FA genes. It affects the same signaling pathway through multiple sets of genes and is highly sensitive to damage of intracellular genetic material (7). Studies have found that the deletion of FA gene is closely related to reproductive diseases, especially interference with primordial germ cells (PGCs), including ovarian and breast cancer in women and azoospermia in men (8–10). Some of the genes in the FA pathway are important for the repair process of ICL of DNA (11). In this review, we focus on exploring the relationship between FA genes and male azoospermia. For example, Krausz et al. (12)reported a high correlation between NOA and the pathogenic variation of the Fanconi gene in the FA pathway; Tsui et al. (13)also suspected that homozygous mutations in *FANCM* can lead to the occurrence of NOA. The association between the Fanconi gene and male NOA deserves further research, such as whether abnormal DNA damage and calcium signaling abnormalities in FA have an impact on sperm formation and maturation (14). Based on the relevant research results in recent years, this review summarizes the molecular-level interaction mechanism and relationship between the specific genes of FA and NOA, aiming to provide helpful insights for the treatment of azoospermia and research on FA from a genetic perspective.

## 2 The mechanism of fanconi anemia

**Figure 1:**
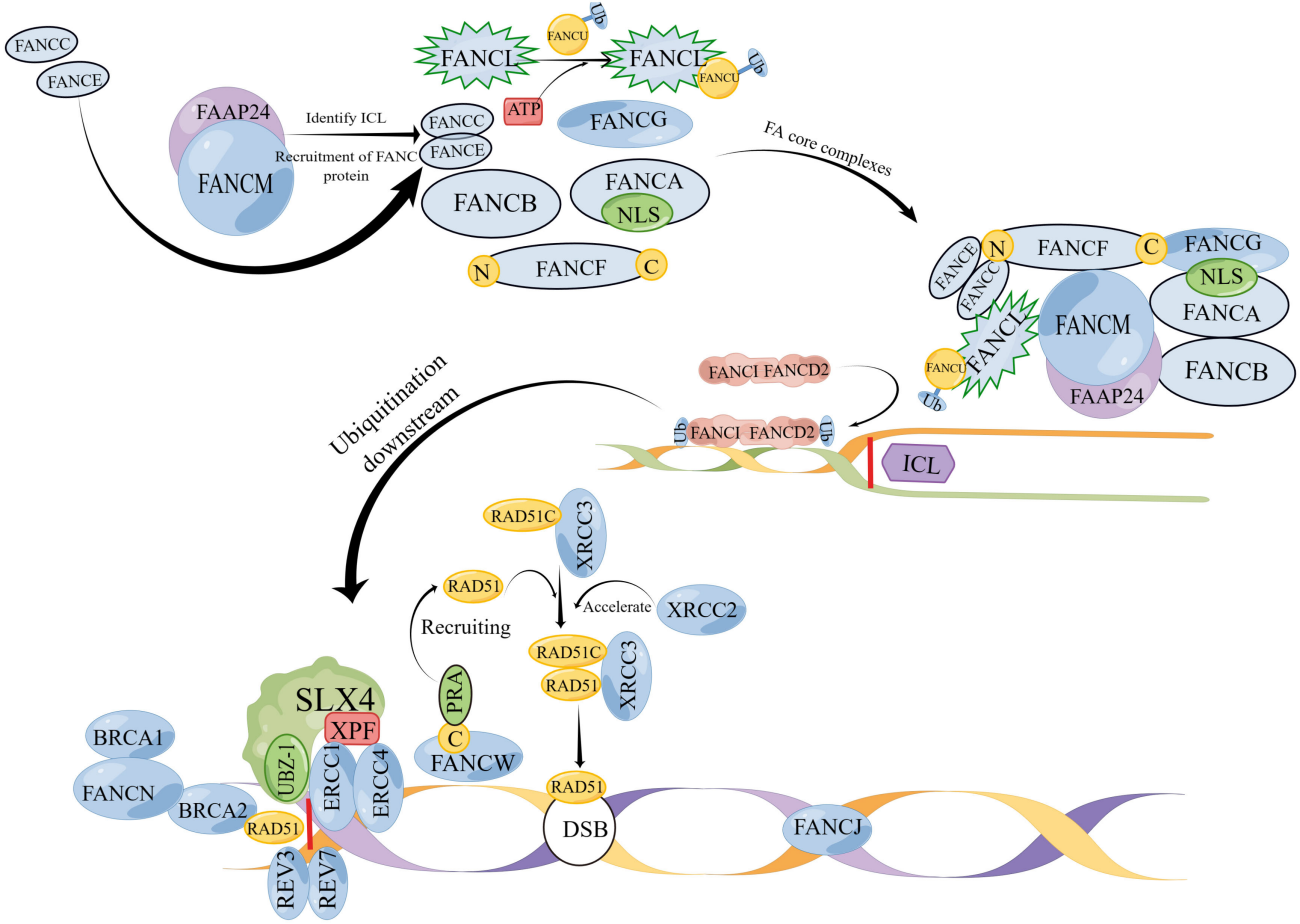
(By Figdraw) A model of the mechanism of action of FA genes. The FA gene action sites are divided into three parts: upper, middle, and lower. Proteins such as FANCA, FANCB, FANCC, FANCE, FANCF, FANCG, FANCI, FANCL and FANCM that exist upstream combine to form FA core complexes; ubiquitination of the ID2 complex located in the middle reaches is the main step in repairing DNA damage such as ICL, and the ID2 complex is the main pathway for activating downstream; the downstream protein SLX4 (FANCP) catalyzes ICL decoupling and binding to ERCC4 (FANCQ) through the UBZ-1 domain. REV7 (FANCV) and REV3 form a DNA polymerase complex for ICL repair. Under the action of FANCN, BRCA1 (FANCS) binds to BRCA2 (FANCD1) and acts together with RAD51 (FANCR) to repair DSB. RAD51C binds to XRCC3 to form a complex and is dependent on RAD51 to maintain normal division.However, XRCC2 (FANCU) forms a complex with RAD51 and has replication restart effect, which can repair homologous recombination. The C end of FANCW binds to PRA and is recruited by it to restart the replication fork. RAD51 (FANCR) can be used to repair DNA double-strand break. FANCJ repairs DNA interstrand cross-linking through homologous recombination (HR). (Note: The diagram is not the actual spatial configuration of the FA core complex, it is only a schematic diagram).

**Figure 2:**
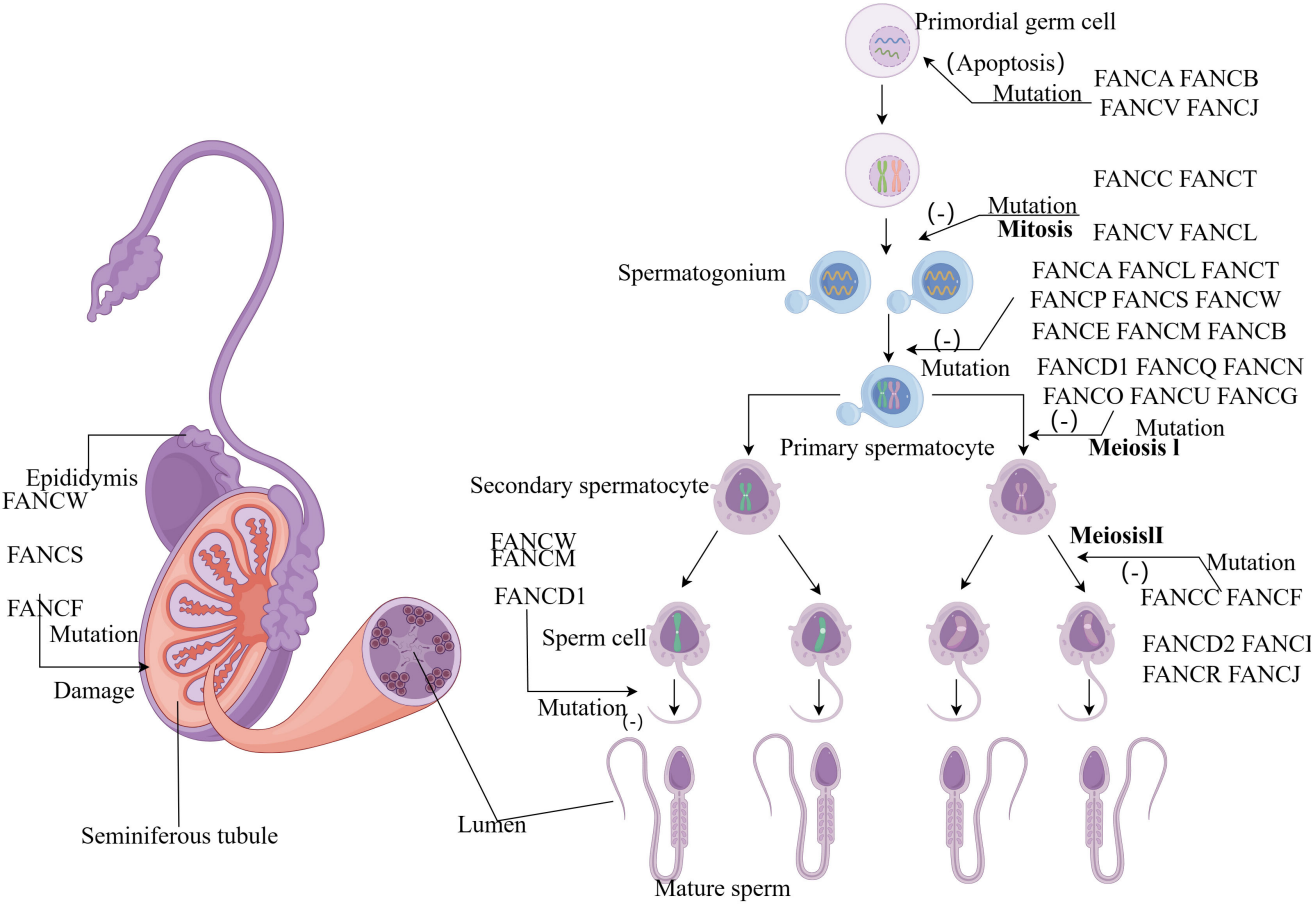
(By Figdraw) Schematic diagram of the influence of FA gene on sperm development process. FA genes affect almost every stage from primitive germ cells to sperm development. Mutations in the *FANCA*, *FANCB*, *FANCV and FANCJ* gene can cause apoptosis in primordial germ cells. During the mitotic stage, *FANCC*, *FANCT*, *FANCV* and *FANCL* mutations prevent the formation of spermatogonia, and a group of genes also play an important role in the formation of primary spermatocytes. Most genes affect the meiotic stage, while *FANCD1*, *FANCM* and *FANCW* mutant spermatocytes cannot form mature sperm.

The FA pathway is associated with genetic disorders that primarily play a role in DNA replication (11). DNA replication includes processes such as unwinding, catalysis by DNA polymerase, and DNA ligase connecting Okazaki fragments, and replication is carried out in a semiconservative replication manner (15). This process requires the collaboration of multiple enzymes and is prone to errors. Damaged DNA needs to be repaired or cleared in a timely manner, and FA signals are involved in this process (15, 16). There are currently 22 known genes related to the FA pathway (16): *FANCA (* 17), *FANCB (* 18), *FANCC (* 19), *FANCD1* (*BRCA2*) (20), *FANCD2 (* 21), *FANCE (* 22), *FANCF (* 21), *FANCG* (*XRCC9*) (17, 23), *FANCI (* 21), *FANCJ* (*BRIP1*/*BACH1*) (24), *FANCL (* 25), *FANCM (* 26), *FANCN* (*PALB2*) (27), *FANCO* (*RAD51C*) (28), *FANCP* (*SLX4*) (29), *FANCQ* (*ERCC4*) (30, 31), *FANCR* (*RAD51*) (31), *FANCS* (*BRCA1*) (32), *FANCT* (*UBE2T*) (25), *FANCU* (*XRCC2*) (33), *FANCV* (*REV7*) (34) and *FANCW (* 35, 36) (**Table 1**). Research has shown that the FA pathway can be classified upstream, middle, and downstream of FANCD2 ubiquitination: Upstream is mediated by a complex enzyme composed of FANCA, FANCB, FANCC, FANCE, FANCF, FANCG, FANCI, FANCL and FANCM (110). The ubiquitination of FANCD2 by these complex results from the activation of FANCT by FANCL, and FANCD2 often binds to FANCI, resulting in the ubiquitination of FANCD2-FANCI (ID2) complex (111–113). The ubiquitination of the ID2 complex induces processes such as cleavage and homologous recombination of endonucleases, leading to de-ubiquitination and ultimately completing DNA damage repair (114, 115) (**Figure 1**). In the FA pathway, signaling networks formed by protein complex enzymes such as FANCA can dynamically monitor the process of DNA changes (116). Damage or termination of DNA replication forks can activate upstream FA proteins, thereby regulating downstream genes such as *FANCP* to correct DNA damage and restore normal replication process (116). One of the most important links connecting the upstream and downstream FA signaling pathways is the ubiquitination of FANCI-FANCD2 (116). The FRT-flanked neomycin cassette can target the *FANCG* gene in mouse embryonic stem cells, causing the defect of this gene and further blocking the protein signal network that binds FANCG to the SH3 domain of RIISp, and the DNA repair of embryonic stem cells cannot be completed (117, 118). Embryonic stem cells can differentiate into germ cells, so blocking the FNACG signaling pathway may reduce the number of sperm in mice (117–119). In addition, exogenous stimuli such as cisplatin and mitomycin C lead to the production of cellular ICL, where the FA protein recognizes and repairs damaged DNA or excises faulty coding (120). When the FA gene is missing, the damaged DNA accumulates in the cell, The resulting self-nucleic acids activate the cyclic GMP-AMP synthase (cGAS) in the cytoplasm to produce secondary messenger cGAMP, which in turn activates type I interferons (IFN-I), which is involved in the emergence of Fanconi related disease phenotypes via the cGAS-STING-IFN-I axis (120, 121). It can be seen that FA, as a signal pathway of target gene, is closely related to DNA damage repair. In addition, studies have shown that abnormal DNA repair can lead to the occurrence of NOA (6). Taking FA signaling pathway as the starting point, we will elaborate on the effects and possible roles of various FA genes in male NOA according to the upstream, middle, and downstream processes of FANCD2 ubiquitination (**Figure 2**).

**Table 1:** The position of human FA gene and its mechanism and influence on NOA.

Site of action	Gene	Another name	Chromosomal location	Mechanism	Impact on NOA after mutation	References
Ubiquitination upstream	FANCM		14q21.2	Regulation of the ubiquitination of FANCD2; S-phase identification ICL; Participation in the formation of replication forks; Repair of damaged DNA	Copy fork stop; ICL repair failed; Sperm maturation arrest; dyszoospermia, Inability to conceive	(37–42)
	FANCA		16q24.3	Accumulation of FANC ubiquitinase, Stable DNA replication;	Apoptosis of PGCs and decrease in sperm count; Persistent double strand breaks in DNA	(43–45)
	FANCG	XRCC9	9p13.3	Formation of a complex with FANCA to assist with FANCA protein	Reduced number of germ cells; Persistent double strand breaks in DNA	(17, 45, 46)
	FANCB	FAAP95	Xp22.2	Enrichment and maintenance of spermatogonia; Repair of DNA damage	Reduced PGCs and infertility; ICL repair failure, termination of HR and SCEs, meiosis failure	(18, 47, 48)
	FANCC		9q22.32	Participation in the ubiquitination process, repair of DNA damage, and germ cell maintenance	Interference with the mitotic process of germ cells	(19, 49, 50)
	FANCE		6p21.3	In the S phase, FANCD2 can be ubiquitinated and participate in the assembly of FA complexes, as well as in the repair of ICL; Formation of FANCE/FANCC complexes to participate in the FA pathway	Few germ cells; Low intracellular levels of FANCC, failed assembly of FA complexes, failed ubiquitination of FANCD2, failed ICL repair, blocked cell division cycle, increased cell mortality, difficulty in spermatogenesis	(22, 51–57)
	FANCF		11p15	Protection of the genome; Maintenance of stability of FA core complex and promotion of ubiquitination of ID2 complex	Increased COs levels; Gonad damage	(21, 58–60)
	FANCL		2p16.1	Repair of damaged DNA; Regulation of ID2 complex, reduction of DNA strand cross-linking, inhibition of DNA fragment crossing, and stabilization of cellular genetics	Massive apoptosis of germ cells, damage to sperm in the epididymis, and failure of normal differentiation of spermatogonia	(61)
	FANCT	UBE2T	1q32.1	Regulation of ID2 complex, reduction of DNA strand cross-linking, inhibition of DNA fragment crossing, and stabilization of cellular genetics	DNA replication disorder, affecting germ cell mitosis	(61, 62)
Ubiquitin	FANCD2		3p25.3	Participate in repairing damaged DNA; Initiation of processes such as nucleotide excision and homologous recombination; Reactivation of cell cycle	The genetics of germ cells are prone to damage during division, resulting in minimal sperm count	(63–65)
	FANCI		15q26.1	Participation in repairing damaged DNA; Initiation of processes such as nucleotide excision and homologous recombination to reactivate the cell cycle	Reduced number of spermatocytes	(63, 64, 66)
Ubiquitination downstream	FANCP	SLX4	16p13.3	Maintenance of gene stability; Repair and inhibition of DNA ICL; Inhibition of intermediates produced by homologous recombination	Increased COs levels	(29, 67, 68)
	FANCQ	ERCC4	16p13.12	XPF/ERCC1 directly acts on the ICL repair site; XPF participates in nucleotide excision repair and indirectly participates in ICL repair; Ensures the correctness of chromosome separation	ICL repair defects, Meiotic recombination error, Sperm cell cycle arrest	(30, 31, 69–74)
	FANCV	REV7/MAD2L2	1p36.22	Formation of DNA polymerase subunits, Participation in repairing damaged DNA	Reduced number of PGCs; Apoptosis of primitive germ cells and underdeveloped testicular germ cells, Cell cycle arrest, chromosome breakage, Difficulty in cell proliferation and spermatogenic defects	(75–80)
	FANCN	PALB2	16p12.12	Participation in homologous recombination repair; Combination of FANCS and recruitment of FANCD1 and FANCR	Chromosome recombination obstruction, cell cycle arrest, Germ cell apoptosis	(27, 81–85)
	FANCS	BRCA1	17q21.31	Participation in DNA damage repair, maintenance of genome integrity and stability	Reduced gene expression related to repairing DNA damage, Inability to repair damaged spermatocytes in a timely manner, reduced testicular volume and inability of the seminiferous tubules to produce sperm	(32, 86)
	FANCD1	BRCA2	13q12.3	Participation in DNA repair through the FA pathway and in homologous recombination repair process through ID2 ubiquitination; Regulation of RAD51 protein participation in homologous recombination repair through binding to DNA	ICL repair failure, meiosis stagnated at checkpoints; Abnormal spermatocyte-to-sperm process, Decrease in the proportion of sperm maturation, cell apoptosis, and male symptoms of oligospermia or azoospermia	(20, 87–90)
	FANCO	RAD51C	17q22	ICL repair; Protection of correct chromosome segregation during meiosis	Enhanced sensitivity of DNA interstrand cross-linking; Suspended development of spermatocytes during the first division of meiosis	(28, 91–94)
	FANCR	RAD51	15q15.1	ICL repair; HR for regulation; Protection of correct chromosome segregation during meiosis	Termination of meiosis leading to azoospermia; Chromosome division error causing crossover (CO) phenomenon, Inaccurate chromosome localization	(28, 91, 92, 95)
	FANCU	XRCC2	7q36.1	Participation in restarting the replication process; Participation in the process of repairing double chain fractures	Copy fork collapse, chromosome breakage, cell division termination; Homologous recombination repair defects, cell apoptosis	(33, 96–99)
	FANCW	RFWD3	16q23.1	Participation in homologous recombination and DNA repair process; Recruitment by RPA to a stagnant replication fork, participation in the ATR signaling pathway through phosphorylation of checkpoint kinase ATM/ATR, and control of DNA replication checkpoints to restart the replication fork	Cell cycle arrest and apoptosis	(87, 100–106)
	FANCJ	BRIP1/BACH1	17q22.2	Participation in repairing ICL and DSB through HR; Regulation of replication forks reduces damage to chromatin structure during replication, ensuring normal chromatin replication while maintaining genetic stability	Reduced resistance to ICL; Severe interference with spermatogenesis	(24, 107–109)

ICL, interstrand cross-linking; PGC, primordial germ cell; HR, primordial germ cell; SCE, sister chromosome exchange; CO, crossover; DSB, double-strand breaks.

## 3 The role of ICL in repairing defects leading to NOA occurrence

In physiological conditions, DNA replication is more common in poorly differentiated embryonic stem cells with the ability to divide (122). ICL is a type of DNA damage that poses great harm during DNA replication, known as the obstruction of replication forks, which has a significant effect on the development of germ cells (123). ICL can terminate normal DNA unwinding, causing replication and transcription abnormalities and leading to meiosis arrest, chromosome breakage, and abnormal chromosome binding, significantly damaging the cell’s proliferative ability and causing abnormal germ cell development (124, 125). Zhang et al. found that the Sertoli cell-only syndrome phenotype of NOA may be related to ICL repair defects and is associated with DNA replication in germ cells (126). In the upstream of the FA signaling pathway, mutations in the *FANCM/A/G/B/C* gene have been shown to be associated with human NOA (12, 46, 47, 49, 127). Among them, FANCM defect will cause the FA core complex enzyme in the cell to be unable to recognize ICL in time, and the deletion of FANCA/G/B/C gene will cause the FA core complex enzyme to be unable to associate with FANCD2 ubiquitination, resulting in abnormal repair of ICL (17, 18, 37, 50). ICL persists and interferes with the normal replication of DNA, eventually leading to the proliferation of male germ cells and a decrease in male sperm count (124, 125). The ubiquitination of FANCD2 is closely related to the repair of ICL, and *FANCD2* mutations occur at the K561 site in patients with FA. Due to genetic mutations, FANCD2 is unable to complete ubiquitination (51). Failure of the FANCD2 ubiquitination process causes cells to stall in the S phase (51), unable to produce mature sperm. At the same time, in the downstream of the FA pathway, homologous recombination mediated by FANCN/FANCU and other proteins is involved in the subsequent ICL repair process, and the defects of related genes lead to the reduction of sperm quantity and quality (27, 51, 81, 95, 96). The essence of NOA is the reduction of sperm count, which is often caused by the failure of migration of PGCs (128) and abnormal mitosis and meiosis of PGCs (8). For men, a defect in the repair of DNA cross-linking between strands during the formation of sperm can cause problems in the gene expression of PGCs, leading to defects in the sperm and even the inability to form mature sperm, leading to NOA or oligospermia (6). Therefore, the FA signaling pathway is related to the occurrence of NOA, and the defects of related genes will hinder the repair process of ICL and eventually lead to the occurrence of NOA.

## 4 FA gene leads to the occurrence of NOA

### 4.1 The effect of ID2 ubiquitination upstream defects on NOA

#### 4.1.1 FANCM

The expression product of *FANCM* is a protein comprising the FA core complex. The FANCM protein has seven domains: aa 5-12, aa 77-590, aa 661-800, aa 826-967, aa 1218-1251, aa 1818-1956, and aa 1971-2030. Each domain is involved in different stages of FA and has genetic significance for the formation of NOA (26, 129). According to reports, *FANCM* mutants are present in patients with NOA (129). Kherraf et al. analyzed 151 genes using whole-exome sequencing and found a homozygous mutation in the *FANCM* gene of patients with NOA (NM_020937.4: c.5791C>T; p.Arg1931Ter) (127). Previous studies have shown that after truncated *FANCM* mutations, the major histone fold binding domain, MM1 (aa 826-967) domain, MM2 (aa 1218-1251) domain, and ERCC4 (aa 1818-1956) domain cannot express the corresponding proteins, leading to spermatogenesis disorders and infertility (38, 39). Furthermore, FANCM can regulate the ubiquitination process of FANCD2 and play an important role in DNA repair. Studies have shown that mutations in the DEAH (aa 77-590), MM1 (aa 826-967), and FANCM-PIPbox (aa 5-12) domains of FANCM can lead to the inability to recruit FA complexes and FANCD2 to undergo ubiquitination, leading to the disruption of DNA repair processes (38, 40, 41), thereby blocking the proliferation of germ cells. Yin et al. found that adult mouse sperm inside the testicles with *FANCM* mutations were lost and sperm maturation was delayed (42), which may be related to DNA damage, such as FANCM binding to FAAP24 protein at the termination of a replication fork and recognition of ICL in the S phase (37). Therefore, during cell division, *FANCM* mutations may lead to the formation of FA complexes and the inability of FANCD2 to complete ubiquitination, resulting in the inability to repair ICL. Ultimately, the number of sperm in seminiferous tubules may decrease because of the inability of spermatogonia cells to proliferate and mature, leading to the occurrence of NOA.

#### 4.1.2 FANCA-FANCG

The *FANCA* gene is currently one of the genes generating the most interest (43), along with *FANCG*, also known as *XRCC9 (* 23). *FANCA* is an important gene for repairing DNA breakage and is mainly expressed in the testes (130). The complex composed of two gene-encoded proteins is one of the main elements involved in the formation of ubiquitination complex enzymes (17). FANCA uses the amino terminal nuclear localization signal to bind to the FANCG protein to increase the content of the FANCA protein, leading to the accumulation of FANC ubiquitinase in the nucleus (44), which is conducive to stable DNA replication. In contrast, the FANCG protein can assist the FANCA protein in DNA repair (17). Krausz et al. found that pathogenic mutations in *FANCA* lead to NOA (12). They sequenced two types of patients and found that the first type had c.2639G>A mutations, while the second type had two types of mutations, [NM_000135.2: c.3788-3790delTCT, p.Phe1263del] and [NM_000135.2: c.3913C>T, p.Leu1305Phe], both of which had significant pathogenicity (12). Simultaneously, studies have found that *FANCA* mutated and showed high expression (12) after biopsy of tissues from patients with NOA (131). Furthermore, Wong et al. found that under the action of *FANCA*^tm1.1Hsc^ homozygotes, the number PGCs may decrease due to their inability to survive or divide (43). Simultaneously, the number of errors during meiosis in spermatocytes and apoptosis in spermatocytes increase, ultimately resulting in almost zero sperm count in the vas deferens (43). After mutation of the *FANCG* gene, PGCs are blocked during the S phase, resulting in a decrease in the number of PGCs and can interfere with the migration process of PGCs to the testes through Rac1 (46). After a *FANCA* or *FANCG* defect mutation, double stranded breaks in DNA become difficult to repair (45). The FANCA-FANCG complex can interact with each other, and the phenotype of the double mutation is consistent with that of *FANCA* or *FANCG* single mutation, but with a higher degree of harm (132). From this, it can be seen that the FANCA-FANCG complex has a regulatory effect on DNA interstrand cross-linking repair and can lead to NOA by affecting the cell proliferation cycle, especially the meiosis process of sperm.

#### 4.1.3 FANCB

The *FANCB* gene is currently the only known FA gene located on the X chromosome. However most current research focuses on the relationship between this gene and female ovaries, breasts, and other aspects and the research on male azoospermia is scarce. The effects of FANCB on male reproduction can be attributed to two pathways. The first pathway is for FANCB can accumulate in spermatogonia and participate in the maintenance of undifferentiated spermatogonia (47, 48). Kato et al. found that the number of PGCs in male *Fancb*-mutant mice was significantly reduced compared with that in the control group, and infertility occurred (47). As PGCs decreased, the number of spermatogonial cells that divided into spermatogonia also decreased, leading to a decrease in sperm count and the occurrence of NOA. The second pathway is that FANCB can participate in DNA damage repair, ensuring normal meiosis. Studies have shown that the FA core complex cannot ubiquitinate FANCD2 because of the mutant *FANCB*, which leads to the failure of ICL repair. This prevents normal homologous recombination (HR) and sister chromosome exchange (SCEs), leading to failure of division (18). Therefore, the *FANCB* mutation decreases the number of spermatogonia in male testes and may reduce the success rate of meiosis by hindering the repair of ICL and meiotic recombination process, inducing NOA.

#### 4.1.4 FANCC

The expression product of the *FANCC* gene exists in the cytoplasm and nuclear compartments (133). As a core complex of FA, the function of FANCC to repair DNA damage can maintain the activity of sperms and ensure their survival (19). FANCC does not change during the cell cycle, but there is a certain degree of dependent regulation (133). Nadler et al. found that mutations in the *FANCC* gene can interfere with the mitotic process of PGCs, and in males, these exhibit infertility (49). FANCC participates in the ubiquitination process of the FA core complex (50). A mutation in this gene will result in the inability of the FA core complex to ubiquitinate, inactivating the FA pathway and preventing it from participating in replication fork stability and cytoplasmic division, which may further lead to the inability of primordial germ cells to undergo mitosis and meiosis. The division of primordial germ cells is impaired, preventing the formation of mature sperm. In experiments in mice, mice with *Fancc* gene mutations experienced a significant decrease in the number of sperms within more than ten days (49). PGCs are precursors of sperm, and a decrease in PGCs indicates a corresponding decrease in the number of subsequently produced sperm (134). Low sperm count leads to a series of male infertility diseases such as NOA.

#### 4.1.5 FANCE

The product of the *FANCE* gene is a member of the FA core complex (50), which is mainly involved in ICL repair. Fu et al. (52) reported very few spermatocytes in the *Fance* mutant mouse model, and that spermatocytes are directly related to spermatogenesis. Therefore, similar to NOA, *Fance* deficiency significantly reduces the sperm count, leading to impaired reproductive function and infertility symptoms in male mice (52, 135). Research has shown that FANCE participates in ICL repair through both direct and indirect pathways. In the direct pathway, FANCE can ubiquitinate FANCD2 in the S phase and participate in the assembly of FA complexes (51, 53). Then, FANCD2 locates the DNA damage site and recruits FA protein for ICL repair (51). In the indirect pathway, FANCE and FANCC participate in each other’s nuclear accumulation and form the FANCE/FANCC complex regulated by FANCF (54)to participate in the FA pathway (55, 56). After *FANCE* mutation, FANCC cannot accumulate in the nucleus, FA complex cannot assemble (22), and FANCD2 cannot complete ubiquitination, ultimately leading to ICL repair failure. Data shows that *FANCE* mutations can arrest the cell division cycle (57). Loss of *FANCE* leads to increase cell mortality rate, and cause difficulty in spermatogenesis, often resulting in NOA (52, 57). Simultaneously, cell cycle arrest can affect the development of spermatogonial stem cells (136). As the earliest sperm cells, spermatogonial stem cells are of great significance in male reproduction. Therefore, *FANCE* deficiency can lead to cell cycle arrest, affect spermatocyte and sperm development, and lead to NOA.

#### 4.1.6 FANCF

FANCF mainly exists in the nucleus and is an important component of the core complex that constitutes the FA pathway, playing a protective role on the genome (21). FANCF is an adapter protein and has two relatively stable regions located at the N-terminus and C-terminus. The C-terminal binding of FANCG to FANCF seems more stable, while the FANCC/FANCE complex can bind to the N-terminal of FANCF (54). In this way, FANCF protein tightly binds to other core complex components to maintain the stability of the FA core complex and promote the ubiquitination of the ID2 complex (58). Compared with other Fanconi genes, many mechanisms of *FANCF* are not yet clear. Previous studies have shown that *FANCF* mutations can cause instability in the FA genome, damaging the gonads (59). According to relevant data (137), *FANCF* defects can cause testicular lesions and testicular cancer. Spermatogonia develop into sperm in the seminiferous tubules within the testicles (138) ^(^ 139^),^. Therefore, mutations in *FANCF* can further damage the seminiferous tubules by causing testicular damage and making them unable to provide a place for sperm production, resulting in immature sperm development and ultimately NOA. Furthermore, Mutations in *FANCF* protect the crossover phenomenon of DNA, making it difficult to repair its function and adversely affecting fertility (140). Therefore, the deletion of the *FANCF* gene not only causes testicular lesions, causing difficulty in spermatogenesis, but also affects seminiferous tubules, leading to NOA.

#### 4.1.7 FANCL/FANCT (UBE2T)

*FANCL* encodes the FA core complex protein E3 ubiquitin ligase (141), and its encoded product plays an important role in DNA damage repair. *FANCT*, also known as *UBE2T*, encodes E2 ubiquitin-conjugating enzyme (142). The complex formed by FANCL/FANCT (E2-E3) (25) is the only known complex that directly regulates FANCD2-FANCI (ID2) and positively affects the ubiquitination of ID2 complexes. This complex can reduce the DNA interstrand cross-links to a certain extent, inhibit DNA segment crossovers that occur in some cells during DNA replication, and ensure the stability of cellular genetics (61). FANCD2, as a key factor in activating the FA pathway, appears around the nucleus of S-phase PGCs in normal development, indicating a correlation between the FA pathway and fertility (62). Although there are not many reports on these two genes in NOA diseases, we can speculate based on the above research results that *UBE2T* mutation or knockout will result in disorder in the PGCs in DNA replication and also affect normal mitosis (62), and *FANCL* mutations may cause apoptosis in a large number of sperms in the seminiferous tubules, and resulting in a sudden decrease in sperm. Therefore, both *FANCL* and *FANCT* defects can damage sperm formation and lead to NOA in men (143).

### 4.2 The effect of ID2 defect on NOA

FANCD2 is one of the most conserved members of the FA family, and its mono-ubiquitination is key to activating the FA pathway. FANCD2 can interact with other proteins to repair DNA damage and play an important role in many stages of cell development (144, 145). FANCI is mainly located in the nucleolus and can regulate the transcription of ribosomes, maintaining the relative stability of DNA (146, 147). FANCD2 and FANCI combine to form an ID2 complex, both of which are mono-ubiquitinated substrates (21). ICL cannot be repaired without the activation of the FA pathway, and this depends on the mono-ubiquitination of FANCD2 and FANCI, which activates the downstream FA pathway (148, 149). At the molecular level, the ubiquitinated ID2 complex initiates downstream processes such as nucleotide cleavage and homologous recombination, while at the cellular level, it activates the cell cycle to continue dividing (63). Ubiquitination binds the ID2 complex more firmly in space, thus better protecting damaged DNA from external interference and ensuring normal DNA damage response and repair (64). Moreover, by phosphorylating FANCI with ATR kinase, the ID2 complex can maintain its ubiquitination (150). After participating in the FA pathway, the ID2 complex needs to be immediately de-ubiquitinated, which is mediated by the ubiquitin-specific peptidase USP1. Blocking the de-ubiquitination process will lead to the failure of chromosomal homologous recombination and the inability to produce spermatogenic cells (151). Hypospermatogenesis is the testicular phenotype of patients with NOA (152). Furthermore, in mice with *Fancd2* deficiency, double strand break (DSB) and crossovers cannot be repaired in a timely manner, resulting in genetic information damage to PGCs during division and minimal sperm count (65). Simultaneously, mutated *FANCI* will induce FANCD2 lesions and lead to a decrease in the number of undifferentiated spermatocytes, which may develop into NOA (143). In summary, the ubiquitination, ATM and Rad3-related phosphorylation, and de-ubiquitination processes of the ID2 complex are indispensable. A mistake in any link can seriously affect downstream homologous recombination processes (69), leading to ICL repair failure and affecting the normal development of male testes (66), thereby inducing NOA.

### 4.3 The effect of ID2 ubiquitination downstream defects on NOA

#### 4.3.1 The effect of nucleolysis-related genes on NOA

##### 4.3.1.1 FANCP

*FANCP*, also known as *SLX4*, encodes a large scaffold protein that assists structure-specific endonucleases. This protein is important in maintaining gene stability in repairing various types of DNA damage, such as DNA interstrand cross-links (29). This gene is precisely located in pachytene sex chromatin and is closely related to the genetic stability of sex chromosomes (153). The more stable the inheritance of XY chromatin, the stronger the SLX4 intensity and the more complete the genetic material possessed by the sperm (153, 154). We believe that the probability of NOA occurring in a stable genetic environment will also be minimal. In the process of repairing interchain crosslinks, FANCP decouple the cross-linked portion (67). FANCP (SLX4) acts as a scaffold protein and acts on DNA strand cross-linking through the UBZ-1 domain to release the cross-linked portion (68). Simultaneously, this protein can effectively inhibit the intermediate produced by homologous recombination, and SLX4IP regulates the SLX4-XPF-ERCC1 complex, which can, to some extent, inhibit ICL (68). FANCP regulates the correct segregation of chromosomes within PGCs. Furthermore, research has found that the absence of *SLX4* can lead to the abnormal development of spermatogonia, which cannot develop into mature sperm in the seminiferous tubules (139), and the number of sperm decreases. A low number of mature sperm can lead to a low number of sperm in the seminal vesicles, resulting in NOA (155). We speculate that if *SLX4* undergoes mutations or deletions, the meiotic process may be halted owing to genetic instability, resulting in the inability to produce sperm and NOA or oligospermia.

##### 4.3.1.2 FANCQ

*FANCQ*, also known as *ERCC4 (* 156), encodes a protein mainly involved in the repair of ICL. FANCQ plays a role downstream of the FA pathway. XPF expressed through ERCC4 can form a complex with ERCC1, which is recruited by SLX4 with the help of FANCD2 mono-ubiquitination and directly acts on the ICL repair site (30, 31, 69). In contrast, *ERCC4* expresses a specific nuclease XPF that participates in the nucleotide excision repair necessary for ICL repair, thereby indirectly participating in ICL repair (30, 70). *FANCQ* mutations exhibit cell cycle arrest induced by mitomycin C, which may be due to chromosome breakage caused by ICL repair defects (71, 72). Therefore, *FANCQ* mutations may disrupt the process of sperm production or reduce the number of sperm, and hypospermatogenesis and cell maturity arrest are the histological phenotypes of the testicles in NOA (157). Furthermore, FANCQ also plays an important role in the meiotic recombination process. The SHOC1 protein is an ERCC4-like protein, and plays a role in the meiotic process of spermatocytes, promoting the formation of crossovers and ensuring correct chromosome separation to produce haploid gametes, and the ERCC4 protein also has this function (73, 74). Therefore, *FANCQ* mutations may result in chromosome recombination errors during meiosis and arrest the spermatocyte cycle (73, 74). We summarized the possible effects of FANCQ on NOA pathogenesis. FANCQ plays important roles in ICL repair and meiotic recombination. *FANCQ* mutation may prevent ICL repair, cause meiotic recombination errors, and hinder the spermatocyte division cycle, leading to apoptosis, and then lead to hypospermatogenesis often leads to NOA (72).

#### 4.3.2 The effect of TLS-related genes on NOA

##### 4.3.2.1 FANCV

*FANCV*, also known as *REV7*/*MAD2L2 (* 34, 75), encodes a protein that can form a DNA polymerase complex with REV3 (76), enabling FANCV to greatly promote REV3’s ability to repair DNA damage and play an expanding role in ICL repair in the form of enzymes (77, 78). ICL repair is closely related to spermatogenesis, indicating the importance of FANCV in regulating cell mitosis and cell development. Experimental reports show the extreme abundance of REV7 in adult male testes (158). After the deletion of this gene, the number of PGCs in mice decreases and they exhibit infertility (79). Furthermore, studies have shown that after *FANCV* mutation, PGCs undergo apoptosis during the embryonic stage, resulting in spermatogenesis defects and testicular sperms are underdeveloped (75). The structural domain mutations in the interaction between FANCV and REV3 manifest as cell cycle arrest, chromosome breakage, and difficulty in cell proliferation, leading to difficulty in spermatogenesis (80). Therefore, REV7 can maintain the normal morphology and function of sperms, and *FANCV* mutations can lead to sperm maturity arrest, leading to NOA (157).

#### 4.3.3 The effect of homologous recombination related genes on NOA

Homologous recombination is important in chromosomal inheritance and DNA repair during mitosis and meiosis. Homologous recombination maintains genome stability by regulating damaged replication forks or filling DNA gaps, and can be used to repair DNA double-strand break (159, 160). Studies have shown that the FA pathway can also repair DNA damage (161), and many genes in the FA gene family are involved in homologous recombination processes, such as *FANCR* (*RAD51*), *FANCS* (*BRCA1*), *FANCD1* (*BRCA2*), and other genes that encode crucial homologous recombination proteins (162). Homologous recombination repair affects spermatogonial stem cells and may be associated with NOA (163).

##### 4.3.3.1 FANCN

*FANCN*, also known as *PALB2*, encodes a gene product that can participate in homologous recombination repair, which is related to the repair of ICL and the smooth progression of the cell cycle (27). Cell cycle arrest and cell apoptosis often cause NOA. FANCN recruits DNA damage sites by connecting one end of the coil domain to FANCS (BRCA1), while the other end recruits FANCD1 (BRCA2) and FANCR (RAD51) to DNA damage sites to initiate homologous recombination repair (82). Furthermore, phosphorylation of FANCN by the cell cycle checkpoint regulatory factor ataxia telangiectasia mutated can promote the repair process of homologous recombination by RAD51 (164, 165). The n-terminal mutation of FANCN prevents homologous recombination repair involving FANCN (81, 83). According to Simhadri et al., removing the *Palb2* gene in mice can lead to a significant reduction or apoptosis of male sperms, affecting fertility (81). Defects in homologous recombination repair of cells can hinder chromosomal recombination during decelerative division, and spermatocytes exhibit cell cycle arrest (81). Male spermatogonial stem cells will gradually develop into sperm (166), and in mice lacking *Palb2*, the number of spermatogonial stem cells significantly decreases. We speculate that the number of male sperms lacking this gene will also show a downward trend, resulting in a sudden decrease in the number of sperm formed in the future. Therefore, FANCN mutations may be potential risk factors for NOA.

##### 4.3.3.2 FANCS

*FANCS*, also known as *BRCA1 (* 167), encodes a protein that is important in DNA damage repair and transcriptional regulation (168, 169), and can also maintain genomic integrity and stability by participating in cellular DNA damage repair (32). Research has shown that BRCA1 can interact with Werner syndrome protein to jointly repair ICL (170). According to Durocher et al., the *BRCA1* gene is present in interstitial cells of the testis, and *BRCA1* deficiency can lead to impaired expression in interstitial cells of the testis (171). Interstitial cells of the testis play a controlling role in male fertility, and if damaged, they can lead to a decrease in sperm quality and quantity (172). Therefore, mutations in *BRCA1* damage interstitial cells of the testis, further affecting sperm count and indirectly leading to NOA. Furthermore, research has found that when *FANCS* mutates, the testicular volume significantly decreases, the seminiferous tubules cannot produce sperm, and the expression of genes related to the repair of DNA damage is reduced, making it difficult to repair damaged spermatocytes in a timely manner (86). Sciurano et al. found that spermatocyte division was stagnant and spermatocyte death occurred in a patient with azoospermia (173). Therefore, patients with *FANCS* mutations are likely to further develop NOA due to irreversible damage to spermatocytes.

##### 4.3.3.3 FANCD1

*BRCA2*, also known as *FANCD1*, encodes a protein that regulates the RAD51 protein by participating in ID2 ubiquitination and binding to DNA, thereby participating in homologous recombination repair processes (87, 88). BRCA2 can control the stability of the genome while maintaining normal meiotic processes, playing an important role in the maturation and development of sperms (174). Studies have shown that BRCA2 is associated with male azoospermia and plays a controlling role in meiosis (89). *BRCA2* deficiency can lead to a meiotic disorder in spermatocytes, leading to infertility and directly affecting spermatogenesis, leading to male azoospermia or oligospermia (89). Shukron et al. compared the structure of male fruit flies with *Brca2* homozygous and heterozygous deletions, as well as wild-type fruit flies, and found that male fruit flies with homozygous mutations experienced sperm arrest, testicular development abnormalities, and infertility (175). Shive et al. found oligospermia in male zebrafish in a homozygous mutation model of *Brca2 (* 176). Simultaneously, a report showed that homozygous deletion of *FANCD1* resulted lower sperm maturation (90). Therefore, mutated *FANCD1* may lead to the failure of ICL repair, meiosis stagnation at the S-phase checkpoint, failure of the spermatocyte-to-sperm process to proceed normally, gradual apoptosis, and oligospermia or aspermia (20, 87, 89).

##### 4.3.3.4 *FANCO* and *FANCR*

*FANCO* is also known as *RAD51C*, and *FANCR* is also known as *RAD51 (* 177). FANCO, a newly isolated member of the RAD51 (FANCR) family, shares genetic similarities with other members. Therefore, RAD51C is a collateral homolog of RAD51 (178). Both play important roles in repairing DNA interstrand cross-links. In DNA damage, overexpression of the RAD51 paraprotein XRCC3 increases resistance to ICL inducers, while RAD51C binds to XRCC3 to form a complex dependent on RAD51 to ensure proper chromosome separation during meiosis (28, 91, 92). Mutation or deletion of *RAD51C* can enhance the sensitivity of cross-linking between DNA strands (93), and RAD51C can transmit DNA damage signals to induce DNA damage repair (179). Some studies have found that *RAD51* mutants develop azoospermia due to meiosis termination (95). Simultaneously, in a mouse model, Qin et al. found (180) that the knockout of *Rad51* led to abnormal development of spermatogonocytes, a reduced number of pachytene spermatocytes, or apoptosis. If defective *RAD51* results in a reduced sperm count, NOA is further increased. Similarly, mutations in *RAD51C* can induce the suspension of spermatocyte development, which mainly occurs in the first phase of meiotic division and is related to abnormal chromosome breakage or premature sister chromosome separation (94). The further development of meiotic arrest in spermatocytes may result in NOA (173) Therefore, FANCO and FANCR are interdependent, ensuring normal progression of meiosis. If both mutations occur, they can affect spermatogonia and spermatocytes, causing NOA.

##### 4.3.3.5 FANCU

The alias of *FANCU* is *XRCC2*, and its expression product can form a complex BCDX2 with FANCR (RAD51) homologous compounds (33, 178, 181). This complex plays a role in replication restart in homologous recombination repair (96). *FANCU* mutation reduces the stability of BCDX2 (96), resulting in the collapse of the replication fork due to the inability to restart, resulting in chromosome breakage and forced termination of cell division (97, 98). Sanger sequencing of *Fancu* mutant mice revealed a recessive mutation in *Fancu* (C. 41T>C/p.Leu14Pro), which stopped the meiosis of cells, thus leading to azoospermia (95). BCDX2 complex participates in the formation of RAD51 nuclear protein filament by binding with single-stranded DNA, and plays a role in double-strand break repair in the assembly of RAD51 lesions (33). *FANCU* mutant cells showed defects in homologous recombination damage repair (99). Simultaneously, Zhang et al. used whole-exome sequencing to sequence two patients with infertility and found that male patients with NOA had C. 41T>C (p.Leu14Pro) *XRCC2* mutation, and believed that the occurrence of NOA in the family was caused by *XRCC2* mutation (182). Therefore, we concluded that the mutation of *FANCU* led to the defective assembly of RAD51 lesions, which resulted in the failure of homologous recombination repair and apoptosis (99). The consequence of incomplete spermatocyte division may be sperm apoptosis, resulting in NOA.

##### 4.3.3.6 FANCW

*FANCW*, also known as *RFWD3*, encodes a protein that plays a role in the synthesis of homologous recombination repair DNA (87). After binding to RPA, the C-terminal of FANCW is recruited by RPA to the stalled replication fork, and the protein is phosphorylated by checkpoint kinase Ataxia-telangiectasia mutated (ATM) and Rad3-related (ATR) to participate in the ATM/ATR signaling pathway and control the DNA replication checkpoint to restart the replication fork (100–104). FANCW accelerates the separation of RPA from single-stranded DNA by regulating its ubiquitination (105), while also promoting the recruitment of repair proteins such as RAD51 and RAD52 by RPA, enabling them to jointly locate DNA damage sites and initiate RAD51-mediated homologous recombination repair (35, 36, 183). This may be due to the *FANCW* mutation, which prevents isolation of RPA from the RAD51 lesion, prevents the formation of RAD51 nuclear filaments, and leads to cell cycle arrest (105, 106). Cell cycle arrest greatly affects the development of spermatogonial stem cells, causing them to stagnate, unable to produce spermatogonial cells or sperm, resulting in a decreased sperm count (136). Simultaneously, the interaction between spermatogonial stem cells and Sertoli cells is regulated, and Sertoli cells promote the maturation and development of testicular seminiferous tubules (166). Although there is currently no relevant literature on *FANCW* mutations directly leading to NOA, we speculate that *FANCW* defects can cause low sperm levels and immature spermatogenic tubules, affecting the maturation and development of spermatogonial stem cells and supporting cells, thereby inducing NOA.

##### 4.3.3.7 FANCJ

The *FANCJ* gene is called *BRIP1* or *BACH1 (* 184). The protein encoded by this gene acts DNA helicase and plays an important role in repairing ICL and DNA DSB through HR (24). Studies have found that mutated *FANCJ* seriously affects spermatogenesis (107). Compared with normal cells, cells with *FANCJ* deficiency or lack of FANCJ catalytic activity have an increased sensitivity to DNA ICL (185), while cells with *FANCJ* mutations significantly reduce their resistance to ICL (108), resulting in a significant increase in the number of ICL within cells. Furthermore, FANCJ can control the normal division of chromosomes by regulating the replication fork, reducing the destruction of chromatin structure during the replication process, ensuring normal chromatin replication, and maintaining genetic stability (109). According to experimental studies in mice, FANCJ causes genetic defects or mutations, with only some males exhibiting fertility. However, there is a significant reduction in testicular volume and a sudden decrease in the number of spermatogonial stem cells (107). NOA is characterized by a low sperm level, whereas spermatogonial stem cells are the most primitive spermatogonial cells, which gradually develop into sperm (186). Although no direct evidence currently links *FANCJ* mutations to the occurrence of NOA, based on our research, we speculate that *FANCJ* mutations may cause genetic and DNA disorders in primordial germ cells, preventing the formation of normal sperms and leading to NOA.

## 5 Summary and outlook

The treatment methods for infertility, including for NOA, are not mature internationally and can be roughly divided into auxiliary technologies, such as spermatogonial stem cell transplantation and intracytoplasmic sperm injection (187, 188). The diagnosis and treatment of NOA through the FA pathway has broad research prospects. If activation of the FA pathway fails, it can lead to unstable DNA replication in PGCs, exacerbating their damage and significantly reducing their number during division, leading to NOA or oligospermia in males. Using the FA pathway to repair the ICL and restore the number of mature sperm in the testes of patients with NOA may cure the disease. Mutations in any FA gene may cause irreparable damage to male reproductive function through the FA pathway and may even be fatal. A key step in the FA pathway is successful ubiquitination of the ID2 complex to repair the damage caused by ICL. Activation of the FA pathway controls the rapidly proliferating PGCs to stabilize the replication and transcription of their DNA (8). At present, relatively novel studies mainly focus on gene level changes such as mRNA therapy and lentiviral vector therapy (189, 190). At the same time, adeno-associated virus integration site 1 has also been found to guide *FANCA* to target the binding of DNA missing this gene fragment to achieve changes in cell phenotype (191). In the future, perhaps we can actively encourage genetic testing and premarital physical examination to reduce the birth of NOA children to some extent from the perspective of prevention. For patients who have been clinically diagnosed with NOA, we can use special mRNA or other special carriers to bind the DNA of FA gene defect to make the expression of FA gene complete again, so as to maintain the number of viable sperm in NOA patients. At the same time, the development of FA protein replacement drugs may be of great significance for maintaining the normal function of FA pathway and improving the reproductive defects in NOA patients, and it is expected to become a new therapeutic strategy. This article reviews the correlation between NOA and FA, categorizes them according to the different gene properties, and attempts to elucidate the relevant mechanisms and significance. We hope that this review will be helpful in solving the problems of DNA damage and FA pathway activation failure caused by FA gene mutations and provide new development ideas and concepts for NOA researchers.

## Author contributions

HX: Writing – original draft, Writing – review & editing. YZ: Writing – original draft, Writing – review & editing. CW: Writing – original draft, Writing – review & editing. ZF: Writing – original draft. JL: Writing – original draft. YY: Writing – original draft. ZZ: Writing – review & editing. YQ: Writing – review & editing. KM: Writing – review & editing. JY: Writing – review & editing. XW: Writing – review & editing.
